# Supplementary material for: Mechanisms governing the pioneering and redistribution capabilities of the non-classical pioneer PU.1
Source: Nat Commun. 2020 Jan 21;11:402. doi: 10.1038/s41467-019-13960-2 (PMC6972792; doi:10.1038/s41467-019-13960-2)
Supplement: Supplementary file 7 — Source data [file 41467_2019_13960_MOESM7_ESM.zip › Source_Data/FigureS2/FigureS2B_MotifScanOutput/KmeansCluster4/homerResults/motif46.similar.html]

motif46

## Information for motif46

C
T
G
A
A
C
T
G
G
T
A
C
C
G
T
A
G
A
C
T
C
T
G
A
C
G
A
T
G
T
A
C
A
G
C
T
A
C
G
T
C
G
T
A
A
G
T
C
  
Reverse Opposite:  

C
T
A
G
A
C
G
T
C
G
T
A
C
T
G
A
A
C
T
G
G
C
T
A
A
G
C
T
C
T
G
A
A
C
G
T
A
C
T
G
A
G
T
C
G
A
C
T
  

|  |  |
| --- | --- |
| p-value: | 1e-19 |
| log p-value: | -4.573e+01 |
| Information Content per bp: | 1.751 |
| Number of Target Sequences with motif | 51.0 |
| Percentage of Target Sequences with motif | 1.13% |
| Number of Background Sequences with motif | 71.7 |
| Percentage of Background Sequences with motif | 0.17% |
| Average Position of motif in Targets | 94.6 +/- 49.0bp |
| Average Position of motif in Background | 98.0 +/- 64.4bp |
| Strand Bias (log2 ratio + to - strand density) | 0.2 |
| Multiplicity (# of sites on avg that occur together) | 1.02 |
| Motif File: | file (matrix) reverse opposite |

### Similar de novo motifs found

|  |  |  |  |  |  |  |  |
| --- | --- | --- | --- | --- | --- | --- | --- |
| Rank | Match Score | Redundant Motif | P-value | log P-value | % of Targets | % of Background | Motif file |
| 1 | 0.903 | T A C G G T A C G T C A G A C T C T A G A C G T T G A C A C G T A C G T G T C A | 1e-16 | -37.513157 | 2.06% | 0.69% | motif file (matrix) |
